# Dietary Intake of Essential, Toxic, and Potentially Toxic Elements from Mussels (*Mytilus spp.*) in the Spanish Population: A Nutritional Assessment

**DOI:** 10.3390/nu11040864

**Published:** 2019-04-17

**Authors:** Ángel Rodríguez-Hernández, Manuel Zumbado, Luis Alberto Henríquez-Hernández, Luis D. Boada, Octavio P. Luzardo

**Affiliations:** 1 Toxicology Unit, Research Institute of Biomedical and Health Sciences (IUIBS), Universidad de Las Palmas de Gran Canaria, Paseo Blas Cabrera Felipe s/n, 35016 Las Palmas, Spain; anrodrivet@gmail.com (Á.R.-H.); manuel.zumbado@ulpgc.es (M.Z.); luis.henriquez@ulpgc.es (L.A.H.-H.); luis.boada@ulpgc.es (L.D.B.); 2 Spanish Biomedical Research Centre in Physiopathology of Obesity and Nutrition (CIBERObn), Paseo Blas Cabrera Felipe s/n, 35016 Las Palmas, Spain

**Keywords:** tolerable intake, essential elements, adequate intake, toxic elements, health risk assessment

## Abstract

The levels of forty-three elements were determined in fresh, preserved, and frozen mussels (*n* = 208) with the purpose of evaluating their contribution to the recommended dietary intake of essential elements and their potential risk to Spanish consumers’ health. We found relevant differences in the element content in relation to the mode of conservation of mussels as well as in relation to their geographical origin, brand, or mode of production. According to our estimates, mussels are important contributors to the intake of most essential elements, contributing almost 70% of daily requirements of Se, 30–35% of Mo, Zn, and Co, and around 15% of Fe. At the same time, the pattern of average consumption of mussels in Spain does not seem to imply an excessive risk associated with any of the 36 toxic elements studied. However, it should be noted that, in the high percentile of consumption the exposure to Cd and As may be high, in particular that associated with the consumption of fresh and/or frozen mussels. According to the results of this study, a moderate consumption of mussels can be recommended as a valuable and safe source of trace elements.

## 1. Introduction

Fishery products provide many beneficial nutritional components such as long-chain polyunsaturated fatty acids (PUFAs), high-quality-proteins, essential elements, and vitamins [1,2]. Among them, mussels are considered an excellent source of proteins, and it is estimated that a 100 g portion of mussel meat provides a quarter of an adult’s daily protein need. It is also considered that the consumption of this amount of mussels provides the recommended daily intake of vitamin B_12_ [3]. It has also been reported that mussels are a relevant source of some essential trace elements, such as Se, Fe, and Zn [3,4]. Taking into account all these nutritional facts and summing up that mussels have low cholesterol levels and a low glycemic index, they should be considered a balanced, healthy, and dietary food choice based on its contribution of nutrients. 

However, this mollusk is also a concern because, in addition to beneficial elements, it also has the capacity to accumulate many other toxic or potentially toxic elements [5,6,7,8,9]. In fact, mussels have been widely employed as sentinel organisms in coastal pollution monitoring, in particular in regard to heavy metal contamination [10]. This is because the gill tissue of mussels is particularly rich in metallothionein, and this attribute of mussels therefore constitutes a key interface for the uptake of dissolved metals and their further incorporation into lysosomes and their transport in blood plasma and circulating hemocytes [10]. In the case of mussels, apart from the abovementioned characteristics, it is also important to consider that a good part of them is consumed in the form of canned preserves, as mussels (similar to the other seafoods) are easily spoiled and very prone to oxidation and to developing off-flavors due to wrong handling or incorrect storage. However, very often, canned foods in general are perceived by consumers as low-quality products, which are thought to be possibly produced using lesser quality raw ingredients, and fresh seafood is in general perceived as the healthier alternative to frozen and processed products [11]. One of the consumers’ suspicions of worse food quality has to do with the packaging material and with the possibility that the canned seafood, apart from their own content in heavy metals from the marine environment, may also be contaminated by heavy metals during the canning process [12].

The mussel canning industry is the recipient of two-thirds of the annual Spanish production of mussels [13]. In general, Spanish mussel production is the second largest in the world, after China’s, with an annual production of around 250,000 tonnes/year (14% of the world’s production) [14]. Mussels are also the type of seafood most consumed by the Spanish population, which has about 15% of regular consumers with an average consumption of around 20.5 kg/person/year (about half in children). This amount is divided between the consumption of canned (40%), fresh (50%), and frozen (10%) mussels [13]. This data is very relevant since, when determining the nutrient concentrations in foods, it is important to consider the different ways of preserving them because they can modify the nutritional composition in a very significant way. Therefore, if one wants to be precise in the estimation of the intake of nutrients and/or contaminants through a certain type of food, all possible ways in which said food is consumed should be considered. In the case of mussels, this would represent the estimation of the intake through one of modes of consumption—canned, fresh, and deep frozen. However, although there is abundant scientific literature documenting the levels of essential and toxic elements in mussels, to the best of our knowledge, very few studies have made an exhaustive comparison of their levels according to their mode of conservation [15,16], and none has taken into account this differential content in the dietary intake estimation.

Consequently, this study was conducted to determine the content of forty-three elements (essential and toxic) in all the forms in which mussels are acquired and consumed in Spain (preserved, fresh, and deep-frozen) with the aim of performing an accurate estimation of the contribution of this food to the daily intake of these elements and of performing a risk–benefit evaluation by comparing the estimated daily intake with dietary and toxic reference values.

## 2. Materials and Methods 

### 2.1. Sampling and Collection

In this research, we studied a total of 208 pooled mussel samples. Mussels were randomly purchased between July and August of 2018 from supermarkets and fish markets of the Canary Islands (Spain). We intended to cover the main forms of presentation of this food in the market, but avoided purchasing preparations and processed mussels (with pickles, sauces, etc.). Thus, we analyzed 88 samples of canned mussels (only steamed and preserved in salted water), 80 samples of deep-frozen mussels, and 40 samples of fresh mussels. Each sample for analysis consisted of 4–5 individual mussels of each brand that were homogenized together. Regarding the origin, the samples were from Galicia (Spain), Chile, and New Zealand, according to the following distribution (Table 1): i) the canned samples were from Galicia (*n* = 72, 38 name brands, and 34 store brands) and from Chile (*n* = 16, all name brands); ii) the frozen samples were from Galicia (*n* = 36, 8 of them were certified as organic production), from Chile (*n* = 28), and from New Zealand (*n* = 12); and iii) the fresh samples were all from Galicia (*n* = 40). In the sampling design, we tried to represent all the possible national and international brands available throughout the Spanish territory. Thus, all the samples came from large suppliers that serve the entire nation, and we consider that our results could be extrapolated and made representative of the Spanish market. After the purchase, all the frozen and fresh mussel samples were kept on ice to maintain the cold chain until their arrival to the Laboratory of Toxicology of the University of Las Palmas de Gran Canaria (ULPGC), where they were processed immediately. 

### 2.2. Standards and Elements

We determined the concentration levels of 43 elements in mussels, including the essential elements and those elements more classically studied because of their high toxicity. Additionally, we included a suite of other elements, including 1) the elements in the ATSDR’s priority list and 2) the rare earth elements (REEs) and other minority elements (ME) that are of increasing concern because of their massive employment in the manufacturing of electric and electronic consumer products and therefore are increasingly appearing as emerging environmental pollutants [17,18]. The complete list of elements comprises the following: Ag (silver); Al (Aluminum); As (arsenic); Ba (barium); Be (beryllium); Cd (cadmium); Ce (cerium); Co (cobalt); Cr (chromium); Cu (copper); Dy (dysprosium); Eu (europium); Er (erbium); Fe (iron); Ga (gallium); Gd (gadolinium); Hg (mercury); Ho (holmium); In (indium); La (lanthanum); Lu (lutetium); Mn (manganese); Mo (molybdenum); Nb (niobium); Nd (neodymium); Ni (nickel); Pb (lead); Pd (palladium); Pr (praseodymium); Sb (antimony); Se (selenium); Sm (samarium); Sn (tin); Sr (strontium); Ta (tantalum); Tb (terbium); Th (thorium); Tl (thallium); Tm (thulium); U (uranium); Y (yttrium); Yb (ytterbium); and Zn (zinc). 

Pure standards of elements in acid solution (5% HNO_3_, 100 mg/L) were purchased from CPA Chem (Stara Zagora, Bulgaria). Two standard curves (twelve points, 100–0.005 ng/mL) were made to avoid interferences between elements: a) one using a commercial multi-element mixture (CPA Chem Catalog number E5B8.K1.5N.L1, 21 elements, 100 mg/L, 5% HNO_3_) containing all the essential elements and main heavy metals; and b) other multi-element mixture tailor-made in our laboratory from individual elements (CPA Chem), which contained the REEs and MEs most frequently employed in the high-tech industry [18].

### 2.3. Analytical Procedure

All the ready-to-eat canned samples were manually pooled using a metal-free Teflon mortar until forming a homogeneous mass. The fresh and frozen samples required a shell opening and cooking in their own juice. This was done by steaming them using a domestic food processor (Thermomix^®^, Vorwerk, Wuppertal, Germany) for a period of 10 min, and after this, the samples were processed in the same way as the canned samples.

For the analysis of elements, mussel samples were acid-digested with the aid of a microwave digester (Ethos Up, Milestone SRL, Italy). Briefly, 500 mg of mussel homogenate were weighed into the digestion vessels, and 50 μL of the internal standard solution (Sc (scandium), Ge (germanium), Rh (rhodium), and Ir (iridium) at a stock concentration of 20 mg/mL each) were added. Next, 2.5 mL of concentrated sub-boiling HNO_3_ (65%) and 7.5 mL of Milli-Q water were added to each sample. All samples were digested according to the following program: Step 1: a power (W), temperature (°C), and time (min) of 1800, 100, and 5, respectively; Step 2: 1800, 150, and 5; Step 3: 1800, 200, and 8; Step 4: 1800, 200, and 7. After cooling, the digests were transferred into conic bottom polypropylene tubes and diluted up to 15 mL with Mili-Q water. Finally, an aliquot of each sample was taken for the analysis. Reagent blanks were prepared similarly to the samples, and a reagent blank was included every 14 samples in the analytical batch. 

For the instrumental analyses, we employed an Agilent 7900 ICP-MS (Agilent Technologies, Tokyo, Japan) equipped with standard nickel cones and a cross-flow nebulizer with a Make Up Gas Port (×400 Nebulizer, Savillex Corporation, MN, USA) for all measurements. All the data were acquired and processed with Agilent MassHunter Data Analysis software (version 4.2, Agilent Technologies, Palo Alto, CA, USA). On a daily basis, the ICP-MS was optimized using a tuning solution consisting of a mixture of Cs (cesium), Co (cobalt), Li (lithium), Mg (magnesium), Tl (thallium), and Y (yttrium) (Agilent Technologies, Palo Alto, CA, USA). All measurements were performed in triplicate from each vial. 

The entire/complete procedure was validated prior to its use in the analyses of samples. Recoveries obtained ranged from 87 to 118% for toxic and essential elements. Linear calibration curves were found for all elements (regression coefficients ≥0.998). Instrumental LODs and LOQs were calculated as the concentration of the element that produced a signal that was three and ten times higher than that of the averaged blanks, respectively. The sample LOQs were calculated by multiplying the instrumental LOQ by the dilution factor suffered by the sample during the digestion procedure (1:10 v:v). 

### 2.4. Dietary Intake Estimates, Nutritional and Health Risk Assessment 

For the estimation of the intake of elements, the total consumption of mussels was taken into account. That is, the consumption of each mussel type (g/day) [19] was multiplied by the median values of each element (ng/g fresh weight) in that type of mussel. The total consumption of each element (ng/kg body weight/day) was obtained by adding the individual consumptions obtained for canned, fresh, and frozen mussels. Both average consumers and high consumers (those in the 97.5th percentile (P_97.5_)) were considered, and the estimations were done for two age groups: adults (>17 years) and children (7 to 12 years).

For the estimation of the risk–benefit ratio, the values of estimated daily intake (EDI) of elements for each scenario (average and high consumers) and age group were compared with the reference values. As dietary reference values (in the case of the essential elements, DRVs), the population reference intake (PRI) values as reported by the European Food Safety Authority (EFSA) [20] were used. According to the European standard, the PRI is the equivalent of the recommended dietary allowances (RDAs) in the USA, that is, the daily dietary intake level of a nutrient considered sufficient to meet the requirements of 97.5% of healthy individuals in each life stage and sex group. In those cases in which the EFSA has not reported the PRI, the adequate intake (AI) was employed as the reference value. AI is the average nutrient level consumed daily by a typical healthy population that is assumed to be adequate for the population’s needs. For those estimates of essential elements that exceeded the PRI or AI, the tolerable upper daily intake level (UL) was considered as well. The UL is the maximum level of total chronic intake of a nutrient from all sources judged to be unlikely to pose a risk of adverse health effects in humans [21,22]. As toxic reference values (TRVs), the non-carcinogen tolerable daily intake (TDI) values from the US EPA [23] were employed. No TRV has been established for Pd and Th, so these two elements were excluded from the risk analysis. No official TRV has been established either for the REEs or the other MEs included in this research. However, some authors have proposed a daily allowable intake of 61 µg/kg body weight (bw) for rare earth oxides [24,25], which was certificated from human health surveys in REE mining areas and animal experimental results. We employed this value as the TRV for these elements, considered as a group (sum REEs). 

### 2.5. Statistical Analysis

Descriptive analyses were conducted for all variables. Arithmetic means, standard deviation (SD), medians, and ranges were calculated for continuous variables. To those data below the LOQ but above the LOD, a random value between those two limits was assigned. Those data below LOD were considered as non-detected. 

The normality of the data was tested using both the Kolgomorov–Smirnov test (with Dallal–Wilkinson–Lilie for *p* values), and the D’Agostin–-Pearson omnibus test. As expected, most of the data series did not follow a normal distribution. Consequently, we chose not to assume a normal distribution in any case, and comparisons between the groups were performed using non-parametric tests (Kruskal–Wallis test or Mann–Whitney *U* test). 

We used PASW Statistics v 25.0 (SPSS Inc., Chicago, IL, USA) to manage the database of the study and to perform statistical analyses. Probability levels of <0.05 (two-tailed) were considered statistically significant. 

## 3. Results

### 3.1. Occurrence of Essential, Toxic, and Potentially Toxic Elements in Mussels 

In Table 2 (essential elements) and Table 3 (toxic and potentially toxic elements), we show the descriptive study of the concentrations found in the three types of mussels considered: preserved, fresh, and frozen. For the great majority of elements, there are significant differences in the levels depending on the mode of conservation of the mussels. The exceptions were Mo among the essential elements and Ag, Be, Pd, Tl, and U among the rest of the elements considered in this research.

Zn, Al, and Fe were the most abundant elements in all the types of mussels, although fresh mussels had the lowest levels of Fe and Al and yet the highest levels of Zn. The fact that one of the most abundant elements is As is surprising, since it can be considered that this element is a contaminant and not a constituent of the biology of the mussels. Additionally, in this case, we found important differences, depending on the mode of conservation, with the highest levels being those in non-preserved mussels (fresh and frozen samples).

The mussels also contain relevant amounts of Sr, Cu, and Mn. Additionally, in these cases, we found concentration differences depending on the mode of conservation of mussels, although these are of lesser importance (Table 2). Cobalt is the essential element present at the lowest concentration by far in all the sampled mussels. With respect to Se, although the concentrations seem not to be high, they can be considered very relevant in nutritional terms, as detailed in the following section.

Regarding the Pb, Cd, and Hg, they were detected in practically all the analyzed samples. In particular, the concentrations of Cd and Pb may be considered relatively high (Table 3) in all the three types of mussels, but in general, we found that canned mussels were the ones that presented the lowest concentrations of these toxic elements.

With respect to the rest of the toxic and potentially toxic elements studied, the concentrations can be considered very low in all cases. As also observed for the other elements, the levels tended to be somewhat lower in canned mussels than in the other two types of mussels considered. Two striking exceptions are Al and Sn, since they are two of the metals used in the manufacture of cans. In fact, Sn concentrations, although low in all cases, are of the order of 3-4 times higher in canned mussels than in fresh or frozen mussels (Table 3). In the case of REEs considered individually, the levels are also very low (Table S1). However, it is very striking that when considered as a sum, the levels in fresh mussels are practically seven times higher than those found in the fresh and frozen mussel samples (Table 3). 

In the main body of the article, we decided to present the results obtained according to the type of conservation of the mussels, since we consider that it is one of the factors that most influences the way in which the final consumer will be exposed. However, we have made other types of analysis, considering the origin of production of the mussels, the type of brand (store brands vs. name brands), and the type of production (conventional vs. organic). We present the results of these secondary analyses as supplementary material (Tables S2–S4). Thus, one of the factors that seems to have a decisive influence on the content of both trace elements and toxic elements of the mussels is their geographical origin. In this research, we sampled mussels from three different geographical regions: Galicia (Spain), Chile, and New Zealand, and statistically significant differences were found. Thus, the mussels of New Zealand presented the highest levels not only of Fe, Mn, Mo, Cr, Co, and Ni but also of As, Cd, Hg, Al, Ba, Sr, Th, Tl, and the sum of REEs. This latter case is particularly striking since the levels of these elements in New Zealand mussels is around six times higher than those from the other origins. On the other hand, Galician mussels presented much higher levels of Pb and of Zn, Cu, Ag, Be, and Sn, although in this latter case it should be taken into account that canned mussels from Galician origin are over-represented in this study, given their high presence in the Spanish market (Table S2), and, as said above, Sn content is higher in canned mussels. 

With mussels produced in Galicia, we could also make a comparison between those that were used for canning in store brands and those canned under name brands, and we also found several statistically significant differences between the two types of branding. Thus, store brands presented significantly higher levels of not only 4 trace elements (Fe, Zn, Ni, and Cr) but also of two toxic elements (As and Hg) compared with those of the mussels of name brands (Table S3). Pb levels were also slightly higher in store brands as well, although this difference did not reach statistical significance. On the contrary, name brands presented significantly higher levels of Ag, Ba, Be, Th, and the sum of REEs compared with those of the store brands.

Finally, it is very interesting to note that we also observed that the concentrations of most of the elements (including the toxic As, Hg, Pb, Ag, Ba, and U) were significantly higher in the mussels of conventional production than in those of organic production (Table S4). The only exception was Al, which was significantly more concentrated in organically produced mussels than in conventionally produced ones. Additionally, in this case, we made the comparison between mussels from Galicia (in this case, all of them were frozen mussels), given that among those of other origins or modes of conservation we did not find organic brands in the Spanish market.

According to the legal limits, the only element for which the maximum residue limits were exceeded was Cd (EU-MRL = 1 mg/kg ww [26]). This limit was exceeded in four samples of frozen mussels from Chile, whereas the other four samples of frozen mussels, also from Chile, reached around 90% of this MRL. In relation to Hg, none of the samples exceeded the MRL established in the EU (0.5 mg/kg ww [27]), and all the samples analyzed were well below this value. The most contaminated sample in the whole series barely reached 10% of this legal limit. Regarding the Pb content, all the concentrations were below the established limit (1.5 mg/kg ww [28]), and the most contaminated sample did not even reach the ½ EU-LMR. No legal limits of As nor the rest of the elements studied have been established in the EU for mussels, so we cannot put the levels found for these elements in a legal context. 

### 3.2. Estimated Daily Intake of Essential and Toxic Elements and Risk Assessment

The results of the estimation of the daily intake (EDI) of essential elements are presented in Table 4, and those of toxic elements in Table 5 and Table 6. The estimation can be considered as quite accurate, since the partial contributions of the different types of mussels to the Spanish diet have been taken into account. This is very important, since, as we have seen in the previous section, there are notable differences in the concentrations of elements, depending on how they are conserved, so the exposure should be calculated on the basis of the real consumption of each type of mussels. 

Given that the differences in the consumption data between men and women (or boys and girls) are minimal [19], we considered it unnecessary to include the comparison between sexes and have presented the results for adults and children, in general, without the consideration of sex. It is noteworthy that the normalized daily intakes per kilo of body weight are practically identical in both adults and children, because the annual consumption of mussels is also practically half in this age group (10.4 kg/year in children vs. 20.5 kg/year in adults), but the average weight of children is half as well. Even so, small differences are observed since the proportions of consumption of the different types of mussels vary between children and adults (adults consume more canned mussels than fresh mussels; in children, however, it is the reverse).

As summarized in Table 4, Table 5, and Table 6, we have considered two groups of consumers: those in the average consumption and large consumption groups, considering as such those in P_97.5_. It can be observed that in the latter the exposure to all the elements is approximately three times that in the average consumer, as the mussel consumption is almost triple as well.

As is logical, regardless of the level of consumption of mussels, the exposure to elements in quantitative terms faithfully follows the concentrations found in the mussels, except in the case of arsenic. That is to say, the highest EDIs are those of Zn, Fe, and Al, followed at a distance by Sr, Cu, and Mo. As EDI does not follow the order of concentrations, although in the previous section we saw that As is one of the most abundant elements in mussels, due to the fact that for the EDI we only considered the fraction of inorganic As (estimated at 3.5% as indicated by the EFSA [29]).

**Figure 1:**
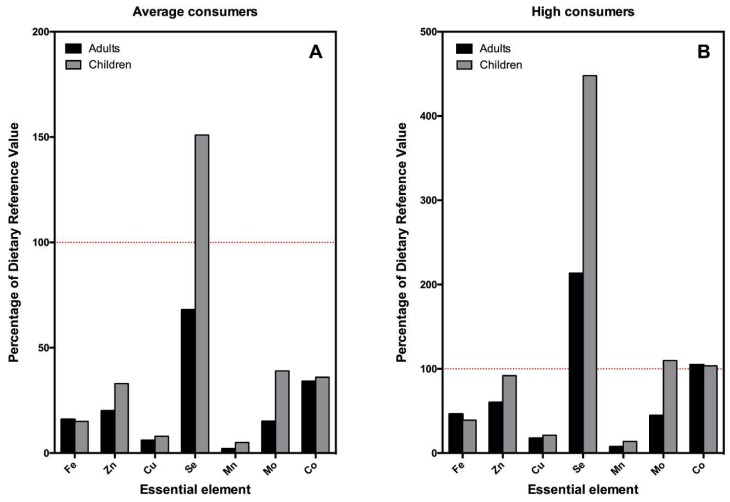
Bar plot indicating the percentage of the dietary reference values (DRVs) of essential elements provided by the consumption of mussels in adults and children. (**A**) Average consumption (percentile 50); (**B**) high consumption (percentile 97.5). Red dotted line indicates 100% of the DRVs of each element. Shaded area between lines indicates.

**Figure 2:**
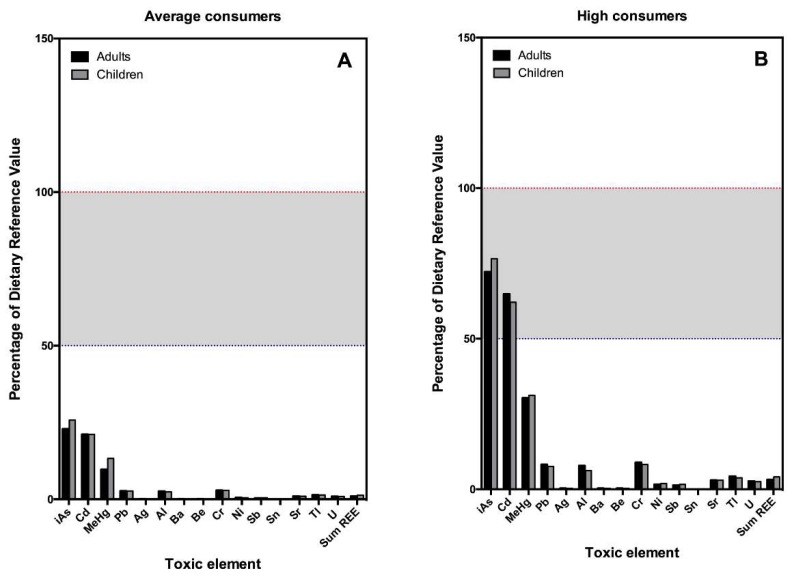
Bar plot indicating the percentage of the TDI of toxic elements provided by the consumption of mussels in adults and children. (**A**) Average consumption (percentile 50); (**B**) high consumption (percentile 97.5). Red dotted line indicates 100% of the TDI of each element. Red dotted line indicates ½ TDI of each element.

However, the most relevant findings arise when the EDIs are compared to dietary or toxic reference levels, which are shown in Figure 1 and Figure 2 (essential and toxic elements, respectively). The average adult consumer of mussels obtains between 2 (Mn) and 70% (Se) of the daily requirements of essential elements through the consumption of mussels. In the case of children, this contribution is superior in all cases. In neither of the two age groups does the average consumption of this food seem to represent a very high risk due to exposure to As, Cd, Hg, or Pb, nor any of the rest of the toxic or potentially toxic elements studied. However, it is important to note that, in children, the levels of exposure to inorganic As and Cd would reach around 26% and 22% of the reference levels, respectively (Figure 1A and Figure 2A). It is also noteworthy that, in those with high mussel consumption, these levels of exposure multiply, reaching almost 77% of the reference values for As in children and 62% of that of Cd (Figure 1B and Figure 2B).

## 4. Discussion

This study represents a very accurate approximation of the risk–benefit relationship of exposure to trace elements through the consumption of mussels in the Spanish population. An exhaustive sampling of mussels has been performed, attempting to reflect all the possible varieties that are acquired by consumers, and a comprehensive determination of the content of elements has been made, covering all the essential elements, a very large group of well-known toxic elements [30], and even those that are currently being considered as potentially toxic or at least emerging contaminants of concern (the REEs and other MEs related to the high technology industry) [18].

Firstly, regarding sampling, our main interest has been to evaluate as closely as possible the exposure of Spanish consumers to elements. Numerous studies have described differences in the levels of elements in mussels due to a multitude of variables. It should be considered that elements in a marine environment have a more complex distribution than organic pollutants and reflect more faithfully local anthropogenic inputs, natural sources, and hydrological conditions [31]. Therefore, one of the most important of these variables is the geographical origin of the said mussels, which in turn is related to the water quality of these regions [32,33,34,35,36,37,38]. However, according to food consumption surveys, geographic origin is not the most important criterion that guides the purchase of mussels by Spanish consumers, but their type of conservation. For this reason, in the design of the sampling, we tried to reflect the different available varieties of the three types of mussels that are chosen by the consumers of this country: fresh, canned, and frozen. 

Very few studies have compared the differences in the element content according to the mode of conservation of mussels [15,16], and as far as we know, none has included these three types of mussels. In the mentioned papers, the authors investigated the differences in the content of some essential (Cu, Mn, Se, and Zn) and toxic (Hg, Cd, Pb, Ag, and As) elements in a wide range of fresh, preserved, and frozen fishery products. However, these authors did not include frozen mussels, and the number of fresh and canned mussel samples studied was very small (*n* = 11 and 12, respectively) [15,16]. The results of these studies showed that there are significant differences in the concentrations of elements of fishery products depending on their mode of conservation, including mussels. Our results confirm that the differences according to the mode of conservation are remarkable in the case of this mollusk, preserved mussels being those that in general have lower concentrations of elements. This is possibly related to a smaller size and age of the mussels that are used in the bulk of the canning industry (12 to 16 medium-size mussels per can is the most usual form of marketing) since most metals bioaccumulate throughout life and lower concentrations are expected in the earlier stages of life (medium-small size mussels). However, regardless of the size, canned mussels have the highest levels of Al and Sn, probably as a result of contamination from the packaging in metal cans containing these elements.

Nevertheless, although we have focused mainly on the conservation mode of the mussels when building our exposure model, other variables such as geographical origin, mode of production, and type of brand were recorded as well. With these variables, we could not force theoretical models of consumption (for example, consumers of only organic products or consumers of only store brands) because not all varieties are available, and therefore they could not all be sampled. However, we would like to highlight some of the results obtained for some of these variables, since such results have never been reported. As far as we know, this study is the first to compare canned mussels of name brands with those of store brands. Surprisingly, although all mussels compared had the same origin (Galicia, Spain), we found significant differences. Mussels of store brands contain slightly higher levels of all the elements (both essential and toxic). This could indicate that mussels of slightly lower quality are used to make these cheaper brands, although the differences are scarcely relevant. The opposite occurs when we compare mussels of conventional production with those of organic production (all of them frozen and of name brands), with the mussels of organic production being those that present lower contents of elements (either essential or toxic). This could be linked to the conditions of the controlled production that organic mussel farming must meet for its certification requirements, which are, for example, a controlled source of the seed, a production unit below 500 rafts and always at a depth of less than 20 m, and the prohibition of some paints [39]. Thereby, more studies should be carried out to understand and confirm those differences since this type of production is in continuous development and economic growth.

According to the exposure estimates for our typical consumer (a Spanish consumer that eats 50% fresh mussels, 40% canned mussels, and 10% frozen mussels), we can summarize that for the average consumption there is a good balance between a moderate to high contribution of trace elements and a moderately low contribution of toxic elements. However, the estimates for toxic elements, in particular As and Cd, can be worrisome, especially in the high percentile of consumption.

Thus, with respect to essential elements, the contribution of mussels to the intake of Se is particularly noteworthy. A regular consumption of this food would contribute almost 70% of the requirements of Se of adults and 150% of those of children. The contribution of Se from mussels is so striking that children who are large consumers would intake up to 500% of their daily nutritional requirements. This contribution would be even higher (more than double) if only fresh mussels were consumed, surpassing around 3–10 times the DRV for this element. However, this contribution is still far from reaching the UL for this element, so it would not pose a real problem of toxicity. This finding is important as Se is a trace element required for different biological functions and is increasingly considered to be a key nutraceutical component. Thus, selenoproteins play a variety of functions, including antioxidant effects, T-cell immunity and implications in the thyroid hormone, and skeletal and cardiac metabolism [40]. In terms of percentage of DRVs, the second element would be Co. The nutritional requirements of Co are not high (of the order of 0.1 µg/kg/day); it is even considered a toxic heavy metal which can cause toxic cardiomyopathy or polycythemia when the exposure to it is excessive [41]. The nutritional contribution of Co is fundamentally associated with the vitamin B_12_ content of the food. Therefore, given that it has been widely described that mussels are rich in vitamin B_12_ [42], it is not surprising that the average consumption of these molluscs represents a contribution of around 40% of the daily requirements of Co. With regard to the rest of the essential elements, mussel consumption is also a high source of Zn, Mo, and Fe. For Zn, a moderate consumption of mussels contributes 20% of the nutritional requirements in adults and up to 35% in children. Zn is a key component of cells and plays a role in the mechanism of action of several crucial enzymes, some of them implicated in the binding of RNA molecules and protein–protein interactions [43]. In the case of Mo, mussel consumption contributes around 15% of the daily requirement in adults and up to 40% in children. This contribution would be even higher (almost double) if only preserved mussels were consumed. Mo is an essential component of certain enzymes that catalyze redox reactions and is also required for enzymes involved in the metabolism of aromatic aldehydes and the catabolism of amino acids [44]. With regard to the contribution of mussels to Fe intake, these account for around 15% of the nutritional requirements in both adults and children. The wide range of roles played by this metal is well known, as it is essential for the maintenance of basic life functions in the form of hemoglobin and is also necessary for electron transfer, oxidase activities, and energy metabolism [45]. On the contrary, mussels represent only a discrete source of Cu or Mn (less than 7% and 4% of their respective DRVs). 

Besides these seven essential elements, the exposure to 36 other toxic or potentially toxic elements through the consumption of mussels was assessed. In general terms, it can be considered that, for the average consumer, mussels pose a very moderate risk of exposure to toxic elements, As, Cd, and Hg being those that reach higher levels. One of the most worrying values is that of the Cd, since, although an average consumer would be exposed to 20% of Cd-TDI, by virtue of the concentrations found, a child consumer who eats only frozen mussels could be exposed to this element so that it reaches up to 40% or even 90%, whether in an average or a high consumer of mussels, respectively. Obviously, this would imply that this type of consumer would greatly exceed the TDI of Cd if the total diet is considered. Something similar happens with As. Our model assumed that 3.5% of the As measured in the mussels is in the most toxic form, the inorganic As [29]. With these estimated values, an average consumer of mussels (whether a child or an adult) would be exposed to approximately 23–25% of the TDI of this element, which, although being relatively high value, is far from representing a real problem of public health. However, it should be noted that mussel consumers in the high percentile would be exposed to up to 75% of the toxicity reference value for this element, and could even surpass it if they only consumed fresh or frozen mussels. The other toxic element that can be worrisome is Hg, as it has also been described in fish [46,47]. Furthermore, in this case, we have made an assumption, which is that 80% of the total measured is in the most toxic form (methylmercury) [48]. In that case, an average consumer would reach around 10% of the TDI, but this value would increase more than three times (>33% TDI) in individuals with high mussel consumption, which is especially worrisome in children, given the special vulnerability of their developing nervous system to the toxic effects of this heavy metal [48]. 

Of the rest of the toxic elements investigated, none exceeds 3% of their respective TDI. Among them, there are elements whose toxicity has been demonstrated, and in fact, they have been included in the priority list published every two years by the ATSDR [30] as elements that are also of increasing concern because of their growing appearance as emerging environmental pollutants, as is the case of the REEs [18]. For many of these, evidence of their toxicity is being provided by many researchers around the world [49,50,51]. Recent studies have shown that mussels can accumulate these kinds of elements as well [8,9] and might even be adversely affected by their presence [52]. In any case, according to the results of this study, neither the REEs nor the toxic elements included in the ATSDR list pose a relevant risk associated with the consumption of mussels.

Finally, it is important to note that, although the risks estimated in this study are not high, in this model we have only considered mussel intake, and this should be taken into account in the global risk assessment. Obviously, mussels are not the only pathway to the exposure to harmful elements in humans, but other relevant foods and other sources, such as soil ingestion, dust inhalation, and dermal contact, should be considered as well. The actual risk is the sum of all the exposure pathways and can be much higher than the ones obtained in this study. 

## 5. Conclusions

For the great majority of elements, there are significant differences in the levels of elements depending on the mode of conservation of the mussels. In general, we found that canned mussels were the ones that presented the lowest concentrations of essential and toxic elements, except Al and Sn, which are two elements employed in the manufacture of cans. We also found significant differences according to the geographic origin, the mode of production, and the type of brand. These are interesting variables, but perhaps they condition less the consumer’s decision when buying mussels (and consequently also their exposure to essential and toxic elements) than they do the mode of preservation of this mollusk. Our exposure model indicates that the average consumption of mussels by the Spanish population (around 20 kg/year in adults and 10 kg/year in children) represents a valuable contribution of essential elements, particularly Se, Co, Zn, Fe, and Mo, without supposing a high risk derived from the exposure to toxic elements. However, there are some worrying aspects, such as the relatively high levels of Cd and As, which might cause high percentages in the reference values for toxicity that could be reached in the case of high consumption of these mollusks. This suggests that a moderate consumption of mussels should be recommended. 

## Figures and Tables

**Table 1:** Sampling of the mussels according to the type of conservation, the production area, the type of brand, and the method of production.

	Galicia				Chile				New Zealand			
	Type of Brand		Mode of Production		Type of Brand		Mode of Production		Type of Brand		Mode of Production	
	Name	Store	Conventional	Organic	Name	Store	Conventional	Organic	Name	Store	Conventional	Organic
Preserved (*n* = 88)	38	34	72	-	16	-	16	-		-	-	-
Frozen (*n* = 80)	36	-	28	8	34	-	32	-	12	-	12	
Fresh (*n* = 20)	40	-	40	-	-	-	-	-	-	-	-	-

**Table 2:** Concentrations of essential in mussels from different types of conservation in the Spanish market. Results are expressed in ng/g.

	Preserved Mussels (*n* = 88)			Frozen Mussels (*n* = 80)			Fresh Mussels (*n* = 20)			*p* Value ^a^		
Element	Mean ± SD	Median	P25–P75	Mean ± SD	Median	P25–P75	Mean ± SD	Median	P25–P75	Preserved vs. Frozen	Preserved vs. Fresh	Frozen vs. Fresh
**Fe**	34,945.1 ± 11,496.8	33,376.0	25,848.3–41,727.6	45,965.2 ± 44,720.5	32,310.0	26,713.1–50,700.1	24,427.6 ± 5553.9	22,924.9	20,137.1–26,850.9	n.s.	<0.01	<0.01
**Zn**	46,011.4 ± 24,034.8	38,961.6	33,105.8–48,439.9	47,141.4 ± 17,788.6	46,231.2	37,356.5–58,914.3	56,624.1 ± 14,019.9	54,712.7	45,823.5–70,423.9	<0.01	<0.005	<0.05
**Cu**	1332.5 ± 383.1	1268.8	1028.4–1492.1	1564.6 ± 340.9	1529.7	1349.5–1831.3	1235.9 ± 195.5	1313.7	1035.5–1405.7	<0.05	n.s.	<0.01
**Se**	646.0 ± 200.8	599.6	538.3–683.3	1075.9 ± 187.9	1085.6	949.0–1229.2	1223.8 ± 125.1	1274.6	1129.5–1315.2	<0.005	<0.005	n.s.
**Mn**	1251.0 ± 261.8	1227.7	1057.9–1407.0	1726.8 ± 812.5	1424.4	1170.0–2079.5	1479.8 ± 298.8	1569.1	1249.4–1702.4	<0.05	<0.01	<0.05
**Mo**	271.4 ± 279.9	151.3	120.3–288.1	187.6 ± 113.6	142.4	106.7–208.5	187.4 ± 45.7	201.9	183.4–216.0	n.s.	n.s.	n.s.
**Co**	40.8 ± 11.0	40.6	33.6–46.5	66.4 ± 67.2	50.2	42.2–75.1	74.2 ± 16.5	74.1	58.9–91.0	<0.01	<0.005	<0.005

^a^ Mann–Whitney *U* test; n.s. non-significant.

**Table 3:** Concentrations of toxic elements in mussels from different types of conservation in the Spanish market. Results are expressed in ng/g.

	Preserved Mussels (*n* = 88)			Frozen Mussels (*n* = 80)			Fresh Mussels (*n* = 20)			*p* Value ^a^		
Element	Mean ± SD	Median	P25–P75	Mean ± SD	Median	P25–P75	Mean ± SD	Median	P25–P75	Preserved vs. Frozen	Preserved vs. Fresh	Frozen vs. Fresh
**Major Toxic Elements**												
**As**	1869.8 ± 634.8	1641.9	1522.5–1973.1	4008.1 ± 59.2	3377.3	3017.5–4992.0	3696.5 ± 432.8	3785.0	3395.5–3994.6	<0.005	<0.005	<0.05
**Cd**	241.9 ± 92.2	232.1	169.1–312.8	463.3 ± 306.4	353.3	252.1–606.6	281.0 ± 89.4	275.3	251.5–335.7	<0.01	<0.05	<0.005
**Hg**	12.4 ± 9.2	11.2	6.4–14.4	14.9 ± 8.6	16.2	6.3–20.6	23.2 ± 3.6	22.5	20.2–26.6	<0.01	<0.001	<0.01
**Pb**	167.5 ± 87.0	184.5	137.3–221.8	176.8 ± 181.0	111.5	21.1–271.9	249.4 ± 64.8	255.9	190.7–296.2	<0.01	<0.001	<0.001
**Other Toxic or Potentially Toxic Elements**												
**Ag**	5.9 ± 2.9	5.4	3.8–7.4	7.6 ± 3.6	7.1	5.1–8.6	6.2 ± 2.3	5.8	4.6–7.5	n.s.	n.s.	n.s.
**Al**	49,278.1 ± 36,202.8	38,878.3	22,886.8–70,388.9	76,417.8 ± 11,1251.9	35,160.6	24,504.6–87,869.5	30,808.6 ± 31,245.7	21,122.7	7570.1–37,324.1	<0.01	<0.001	<0.001
**Ba**	430.0 ± 303.2	318.4	202.8–618.4	708.2 ± 1259.2	353.9	190.0–659.4	238.6 ± 169.6	194.8	138.2–281.2	<0.01	<0.001	<0.001
**Be**	3.0 ± 2.2	2.7	1.1–4.2	3.3 ± 3.9	2.4	0.8–4.3	3.1 ± 1.7	2.8	1.7–4.1	n.s.	n.s.	n.s.
**Cr**	110.4 ± 47.5	101.3	78.0–135.6	109.4 ± 113.6	91.0	61.7–136.4	123.7 ± 28.9	115.5	108.9–129.5	<0.05	<0.05	<0.05
**Ni**	106.2 ± 29.4	103.2	86.4–114.6	140.1 ± 37.5	125.7	95.6–171.9	196.2 ± 59.8	181.1	164.2–235.8	<0.01	<0.001	<0.005
**Pd**	0.07 ± 0.04	0.06	0.04–0.08	0.08 ± 0.02	0.08	0.07–0.1	0.1 ± 0.04	1.1	0.8–4.3	n.s.	n.s.	n.s.
**Sb**	7.3 ± 26.3	1.0	0.05–1.7	1.2 ± 0.5	1.1	0.08–2.1	3.8 ± 1.3	3.5	2.8–4.6	n.s.	<0.01	<0.005
**Sn**	26.2 ± 13.2	24.0	16.7–33.1	9.6 ± 9.3	6.0	1.5–18.0	11.7 ± 6.0	9.6	7.1–15.5	<0.001	<0.001	<0.05
**Sr**	6403.4 ± 3618.4	5764.6	5006.4–6607.25	7169.8 ± 1352.8	6871.1	6293.6–7666.4	7903.9 ± 2478.6	8837.4	6395.1–9575.5	<0.01	<0.001	<0.005
**Th**	7.5 ± 5.9	6.3	2.5–10.8	13.5 ± 24.2	5.5	2.0–13.7	8.3 ± 5.4	6.9	4.6–9.7	<0.05	n.s.	<0.01
**Tl**	1.4 ± 0.6	1.3	0.9–1.8	2.6 ± 2.4	1.8	1.2–2.5	1.3 ± 0.7	1.1	0.8–1.6	n.s.	n.s.	n.s.
**U**	34.5 ± 8.9	33.3	28.5–39.9	36.7 ± 12.3	35.4	29.2–41.8	34.3 ± 14.3	31.8	22.3–48.1	n.s.	n.s.	n.s.
**Sum REE ^b^**	184.8 ± 133.9	144.9	102.1–247.1	330.3 ± 412.1	198.7	155.5–276.2	1913.2 ± 958.3	1570.6	1248.2–2485.42	<0.01	<0.001	<0.001

^a^ Mann–Whitney *U* test; ^b^ This is the sum of the individual concentrations of Ce, Dy, Er, Eu, Ga, Gd, Ho, In, La, Lu, Nb, Nd, Pr, Sm, Ta, Tb, Tm, Y, and Yb; ; n.s. non-significant.

**Table 4:** Estimated daily intake of essential elements through the consumption of mussels by adults and children.

	**Adults (>17 y.o.)—68.48 kg/bw—Both Genders**		
**Essential Element**	**Dietary Reference Value ^a^**	**EDI Average Consumer** **(µg/kg bw/day) ^b^**	**EDI High Consumer (P_97.5_)** **(µg/kg bw/day) ^c^**
**Fe**	160.63 ^d^	24.89	74.41
**Zn**	175.23 ^d^	34.45	105.54
**Cu**	18.98 ^e^	1.09	3.33
**Se**	1.02 ^e^	0.69	2.18
**Mn**	43.81 ^e^	1.06	3.25
**Mo**	0.95 ^e^	0.14	0.42
**Co**	0.12 ^d^	0.04	0.12
	**Children (7–12 y.o.)—34.48 kg/bw—Both Genders**		
**Essential Element**	**Dietary Reference Value ^a^**	**EDI Average Consumer** **(µg/kg bw/day) ^b^**	**EDI High Consumer (P_97.5_)** **(µg/kg bw/day) ^c^**
**Fe**	160.63 ^d^	23.57	62.72
**Zn**	108.06 ^d^	35.34	99.31
**Cu**	14.60 ^e^	1.11	3.10
**Se**	0.51 ^e^	0.77	2.29
**Mn**	21.90 ^e^	1.09	3.05
**Mo**	0.37 ^e^	0.14	0.40
**Co**	0.12 ^d^	0.04	0.12

^a^ For comparison purposes, the DRVs have been expressed in µg/kg·bw/day. ^b^ A consumption of 31.94 g/day of preserved mussels, 22.44 g/day of fresh mussels, and 1.87 g/day of frozen mussels in adults or 11.17 g/day of preserved mussels, 15.96 g/day of fresh mussels, and 1.32 g/day of frozen mussels in children. ^c^ A consumption of 87.98 g/day of preserved mussels, 76.8 g/day of fresh mussels, and 6.4 g/day of frozen mussels in adults or 21.86 g/day of preserved mussels, 52.6 g/day of fresh mussels, and 4.4 g/day of frozen mussels in children. ^d^ Population reference intake is the term employed by the European Food Safety Authority (EFSA) instead of the recommended dietary allowance (RDA) and represents the daily dietary intake level of a nutrient considered sufficient to meet the requirements of 97.5% of healthy individuals in each life-stage and sex group. ^e^ Adequate intake as defined by the EFSA. It represents the amount established that is firmly believed to be adequate for everyone in the demographic group where no recommended dietary allowance (RDA) has been established. When a range of different values for males and females was found, we considered the average value. y.o. years old.

**Table 5:** Estimated daily intake of major toxic elements through the consumption of mussels in adults and children.

	**Adults (>17 y.o.)—68.48 kg/bw—Both Genders**		
**Toxic Element**	**Risk Reference Value ^a^**	**EDI Average Consumer** **(µg/kg bw/day) ^b^**	**EDI High Consumer (P_97.5_)** **(µg/kg bw/day) ^c^**
**iAs ^d^**	0.30	0.07	0.22
**Cd**	1.00	0.21	0.65
**MeHg ^e^**	0.10	0.01	0.03
**Pb**	6.00	0.16	0.49
	**Children (7–12 y.o.)—34.48 kg/bw—Both Genders**		
**Toxic Element**	**Risk Reference Value ^a^**	**EDI Average Consumer** **(µg/kg bw/day) ^b^**	**EDI High Consumer (P_97.5_)** **(µg/kg bw/day) ^c^**
**iAs ^d^**	0.30	0.08	0.23
**Cd**	1.00	0.21	0.62
**MeHg ^e^**	0.10	0.01	0.03
**Pb**	6.00	0.16	0.46

^a^ We have employed as risk reference values (RRVs) the non-carcinogen tolerable daily intake (TDI) values from the US EPA. For comparison purposes, the RRVs have been expressed in µg/kg bw/day. ^b^ A consumption of 31.94 g/day of preserved mussels, 22.44 g/day of fresh mussels, and 1.87 g/day of frozen mussels in adults or 11.17 g/day of preserved mussels, 15.96 g/day of fresh mussels, and 1.32 g/day of frozen mussels in children. ^c^ A consumption of 87.98 g/day of preserved mussels, 76.8 g/day of fresh mussels, and 6.4 g/day of frozen mussels in adults or 21.86 g/day of preserved mussels, 52.6 g/day of fresh mussels, and 4.4 g/day of frozen mussels in children. ^d^ Although in this work we have not made arsenic speciation, we have taken the scientific consensus with respect to arsenic in seafood as a reference, and considered that the inorganic arsenic content in mussels is 3.5% of the As measured. ^e^ We were not able to perform mercury speciation, but it has been established that in fish and seafood the percentage of the methyl form normally seems to vary between 50 and 80%. We were conservatively estimated that 80% of the total mercury in the mussels is in the form of methylmercury. y.o. years old.

**Table 6:** Estimated daily intake of other toxic or potentially toxic elements through the consumption of mussels in adults and children.

	**Adults (>17 y.o.)—68.48 kg bw—Both Genders**		
**Element**	**Risk Reference Value ^a^**	**EDI Average Consumer** **(µg/kg bw/day) ^b^**	**EDI High Consumer (P_97.5_)** **(µg/kg bw/day) ^c^**
**Ag**	5	0.00	0.01
**Al**	1000	26.48	78.21
**Ba**	200	0.22	0.66
**Be**	2	0.00	0.01
**Cd**	3,00	0,09	0,27
**Ni**	20,00	0,10	0,32
**Pd**	N.A.	0.00	0.00
**Sb**	0.4	0.00	0.01
**Sn**	600	0.01	0.04
**Sr**	600	5.77	17.96
**Th**	NA	0.01	0.02
**Tl**	0.07	0.00	0.00
**U**	3	0.03	0.08
**Sum REE**	61	0.59	1.97
	**Children (7–12 y.o.)—34.48 kg bw—Both Genders**		
**Element**	**Risk Reference Value ^a^**	**EDI Average Consumer** **(µg/kg bw/day) ^b^**	**EDI High Consumer (P_97.5_)** **(µg/kg bw/day) ^c^**
**Ag**	5	0.00	0.01
**Al**	1000	24.04	61.99
**Ba**	200	0.21	0.54
**Be**	2	0.00	0.01
**Cd**	3.0	0.09	0.25
**Ni**	20.00	0.11	0.31
**Pd**	NA	0.00	0.00
**Sb**	0.4	0.00	0.01
**Sn**	600	0.01	0.03
**Sr**	600	6.22	18.01
**Th**	NA	0.01	0.02
**Tl**	0.07	0.00	0.00
**U**	3	0.03	0.07
**Sum REE ^d^**	61	0.78	2.51

^a^ We have employed as RRVs the non-carcinogen TDI values from US EPA. For comparison purposes, the RRVs have been expressed in µg/kg bw/day. The REEs have been considered as a sum and compared to the TRV proposed by other authors [24,25]. ^b^ A consumption of 31.94 g/day of preserved mussels, 22.44 g/day of fresh mussels, and 1.87 g/day of frozen mussels in adults or 11.17 g/day of preserved mussels, 15.96 g/day of fresh mussels, and 1.32 g/day of frozen mussels in children. ^c^ A consumption of 87.98 g/day of preserved mussels, 76.8 g/day of fresh mussels, and 6.4 g/day of frozen mussels in adults or 21.86 g/day of preserved mussels, 52.6 g/day of fresh mussels, and 4.4 g/day of frozen mussels in children. ^d^ This is the sum of the individual concentrations of Ce, Dy, Er, Eu, Ga, Gd, Ho, In, La, Lu, Nb, Nd, Pr, Sm, Ta, Tb, Tm, Y, and Yb.

## Supplementary Materials

The following are available online at https://www.mdpi.com/2072-6643/11/4/864/s1. Table S1: Concentrations of rare earth elements and other minority elements in preserved, fresh, and frozen mussels. Median values are provided and expressed in ng/g_fresh weight_. Table S2: Concentrations of essential and toxic elements in frozen mussels from three different production areas. Median values are provided and expressed in ng/g_fresh weight_. Table S3: Concentrations of essential and toxic elements in preserved mussels from name and store brands. Median values are provided and expressed in ng/g_fresh weight_. Table S4: Concentrations of essential and toxic elements in frozen mussels produced under two different production methods. Median values are provided and expressed in ng/g_fresh weight_.
