# Supplementary material for: Dietary Intake of Essential, Toxic, and Potentially Toxic Elements from Mussels (Mytilus spp.) in the Spanish Population: A Nutritional Assessment
Source: Nutrients. 2019 Apr 17;11(4):864. doi: 10.3390/nu11040864 (PMC6521244; doi:10.3390/nu11040864)
Supplement: Supplementary file 1 [file nutrients-11-00864-s001.pdf]

Supplementary Table 1. Concentrations of rare earth elements and other minority elements in mussels from different types of conservation in the Spanish market. Median values are expressed in ng/g fresh weight

	Preserved mussels (n=88)	Frozen mussels (n=80)	Fresh mussels (n = 20)
Ce	49,2	35,8	460,3
Dy	4,0	4,8	0,0
Er	1,8	2,6	19,5
Eu	0,0	4,0	13,7
Ga	7,4	21,0	87,1
Gd	5,8	7,2	66,1
Ho	0,8	1,0	0,0
In	0,0	0,2	0,0
La	28,9	21,4	297,1
Lu	0,0	0,5	2,0
Nb	3,2	3,9	29,7
Nd	24,6	22,5	253,4
Pr	6,3	5,0	61,0
Sm	6,3	5,3	54,8
Ta	0,0	0,3	0,0
Tb	0,0	3,8	9,4
Tm	0,0	1,1	2,5
Y	0,0	49,7	266,6
Yb	0,0	4,4	12,8

Supplementary Table 1. Concentrations of essential and toxic elements in mussels from three different production areas. Median values are expressed in ng/g fresh weight

	Production area (n=80)			
	Galician samples (n=36)	Chilean samples (n=32)	New Zealander samples (n=12)	*P value*[a]
Essential elements				
Fe	36085.4	30485.1	55467.0	<0.005

Zn	50710.0	47888.5	21337.5	<0.005
Cu	1644.0	1584.3	1021.3	<0.005
Se	1100.4	1116.9	861.0	<0.05.
Mn	1341.5	1461.9	2188.7	<0.005
Mo	179.7	115.8	346.6	<0.005
Co	46.8	53.4	144.1	<0.005
Major toxic elements				
As	3113.9	4484.5	6280.9	<0.005
Cd	283.8	535.4	587.0	<0.005
Hg	17.7	5.9	21.7	<0.005
Pb	266.8	18.9	77.7	<0.005
Other toxic or potentially toxic elements				
Ag	6.4	4.6	6.2	<0.05
Al	35882.4	26143.8	83801.8	<0.005
Ba	308.1	184.8	616.2	<0.005
Be	3.5	0.7	3.2	<0.005
Cr	117.4	58.8	141.4	<0.005
Ni	121.8	101.7	181.7	<0.005
Pd	0.1	0.1	0.1	n.s.
Sb	1.0	1.4	1.4	n.s.
Sn	16.9	5.5	5.4	<0.01
Sr	6999.0	6216.4	7649.2	<0.05
Th	7.4	1.5	10.4	<0.005
Tl	1.3	1.2	3.6	<0.05
U	32.0	38.8	26.4	n.s.
Sum REE [a]	277.5	143.2	1498.8	<0.005

[a] This is the sum of the individual concentrations of Ce, Dy, Er, Eu, Ga, Gd, Ho, In, La, Lu, Nb, Nd, Pr, Sm, Ta, Tb, Tm, Y, Yb

Supplementary Table 2. Concentrations of essential and toxic elements in preserved mussels from name and store brands. Results are presented as median values and expressed in ng/g $_{\text{fresh weight}}$

	Name brands [a] (n=38)	Store brands [a] (n=34)	*P value*[b]

Essential elements			
Fe	30671.1	35834.1	<0.05
Zn	37463.5	45988.8	<0.05
Cu	1200.9	1283.0	n.s.
Se	599.6	653.9	n.s.
Mn	1145.5	1249.1	n.s.
Mo	151.3	223.3	n.s.
Co	41.2	43.6	n.s.
Major toxic elements			
As	1632.5	1969.1	<0.01
Cd	224.2	224.5	n.s.
Hg	11.2	12.5	<0.05
Pb	181.0	209.6	n.s.
Other toxic or potentially toxic elements			
Ag	6.6	5.1	<0.05
Al	33950.0	33383.4	n.s.
Ba	335.9	248.5	<0.01
Be	3.3	1.0	<0.01
Cr	94.9	116.5	<0.05
Ni	94.2	111.6	<0.005
Pd	0.1	0.1	n.s.
Sb	1.4	1.0	n.s.
Sn	15.3	15.7	n.s.
Sr	7297.9	6116.6	n.s.
Th	7.3	2.7	<0.01
Tl	1.3	1.2	n.s.
U	33.7	34.2	n.s.
Sum REE [c]	419.5	155.2	<0.005

[a] This comparison was only possible between brands from Galician origin. because we could not find store brands from other origins

[b] Mann-Whitney test.

[c] This is the sum of the individual concentrations of Ce, Dy, Er, Eu, Ga, Gd, Ho, In, La, Lu, Nb, Nd, Pr, Sm, Ta, Tb, Tm, Y, Yb

n.s.: not significant.

Supplementary Table 4. Concentrations of essential and toxic elements in frozen mussels produced under two different production methods. Median values are expressed in ng/g $_{\text{fresh weight}}$

	Conventional production (n=28)	Organic production (n=8)	*P value*[a]
Essential elements			
Fe	36439.6	28740.1	n.s.
Zn	60867.4	38803.3	< 0.01
Cu	1600.4	1714.9	n.s.
Se	1034.4	1168.3	n.s.
Mn	1344.4	1051.4	<0.05
Mo	186.8	153.8	<0.05
Co	47.4	44.4	n.s.
Major toxic elements			
As	3301.7	2288.2	<0.005
Cd	282.8	290.9	n.s.
Hg	18.0	16.2	<0.05
Pb	272.5	235.7	<0.05
Other toxic or potentially toxic elements			
Ag	9.4	7.1	<0.05
Al	58040.4	75981.3	<0.01
Ba	590.6	443.3	<0.05
Be	4.4	4.9	n.s.
Cr	117.4	104.1	n.s.
Ni	123.8	110.4	n.s.
Pd	0.1	0.1	n.s.
Sb	1.4	1.4	n.s.
Sn	18.8	20.4	n.s.
Sr	7218.9	7496.7	n.s.

Th	16.9	18.9	n.s.
Tl	1.7	1.9	n.s.
U	40.9	32.8	<0.05
Sum REE [a]	272.4	279.2	n.s.

[a] This is the sum of the individual concentrations of Ce, Dy, Er, Eu, Ga, Gd, Ho, In, La, Lu, Nb, Nd, Pr, Sm, Ta, Tb, Tm, Y, Yb
